# A case for lifelong learning in emergency medicine: The perspective from a rural state

**DOI:** 10.1002/aet2.10860

**Published:** 2023-03-26

**Authors:** Skyler Lentz, Ashley Weisman, Jordan Ship, Matthew S. Siket

**Affiliations:** ^1^ Department of Emergency Medicine The Robert Larner M.D. College of Medicine at The University of Vermont Burlington Vermont USA; ^2^ Elizabethtown Community Hospital Emergency Department Elizabethtown New York USA; ^3^ Elizabethtown/Ticonderoga Community Hospital Emergency Departments Ticonderoga New York USA; ^4^ UVM Emergency Medicine Residency Program Burlington Vermont USA

**Keywords:** emergency medicine education, lifelong learning, rural emergency medicine, self‐assessment, tele‐medicine

The expansive practice of emergency medicine cannot be fully taught or learned in 3 or 4 years. Lifelong learning, self‐assessment,^1^ and ongoing skill acquisition and retention are necessary. How do we teach and acknowledge this during residency training and how do we support our isolated, rural colleagues? Importantly, there is a growing shortage of rural emergency physicians and many of our trainees may work in these environments.^2^ As educators, we must study potential solutions and bridge these gaps for our trainees entering independent practice and for our colleagues far removed from formal training.

Recent cases, and many like them, illustrate this point. A rural emergency physician expertly places a small‐bore chest tube for an atraumatic pneumothorax, the pneumothorax resolved, they requested transfer to a larger center for ongoing management. The tertiary care center is at full capacity. A phone consultation with a specialist recommends removal of the chest tube in the emergency department (ED) and discharge home if a follow up chest x‐ray shows sustained resolution. The emergency physician has never removed a chest tube before and the patient is transferred to a larger regional ED for uncomplicated removal. In a second case, a patient is transferred from an hour away, during a snowstorm, for a peritonsillar abscess. The procedure is within an emergency physician's scope of practice. The referring physician had never performed this procedure in their decades of practice. The patient is transferred to the tertiary care center where it is drained by the attending emergency physician and emergency medicine resident. On a winter night, an emergency physician intubates a patient with a head injury in their critical‐access emergency department. They request lifesaving medications and the respiratory therapist to set the ventilator. It is nighttime; there is no respiratory therapist or pharmacist. The physician must assist in mixing the medications and setting up the ventilator. The skills required for an emergency physician are ever expanding, particularly as access to tertiary care becomes more difficult. Lifelong learning and practice development to best serve our patients is a necessity in our specialty.

Solutions may include regional conferences, collaborative case reviews, real‐time peer‐based decision support with telemedicine, maintenance of board certification, asynchronous, self‐directed learning through free open‐access medical education (FOAM), podcasts, textbooks or journal articles, and academic–rural partnerships that create shared faculty positions between rural and tertiary sites and rural rotations for trainees. Each of these has its limitations and time represents a major barrier.^3^, ^4^, ^5^ The American Board of Emergency Medicine requires continuous learning for maintenance of certification; this is beneficial but is not tailored to the physician's practice environment.^6^, ^7^

Our own work in the rural state of Vermont and upstate New York has led us to offer high‐acuity, low occurrence (HALO) courses at the regional academic center twice a year for both residents and practicing emergency physicians within our region and create a rural‐specific simulation lab at one of our critical‐access sites. Additionally, we established a tele‐emergency medicine program to offer a virtual resource from the tertiary care center in times of high‐acuity, high‐complexity care, or volume surge. The skill of delivering and accepting tele‐emergency medicine assistance must also be learned and taught. Our education must continue to adapt to meet the needs of our unique and evolving practice environments. We must also acknowledge that many scenarios cannot be predicted. Instilling the motivation for lifelong learning, self‐assessment^1^ and exposure to resource limited settings during training may help prepare trainees for these challenges. Although these issues are not unique to emergency medicine, we are a specialty that prides itself on adaptability. We must confront this challenge with creative solutions.

## AUTHOR CONTRIBUTIONS

Skyler Lentz, Ashley Weisman, Jordan Ship, and Matthew S. Siket conceived the idea for this manuscript and contributed substantially to the content, design, writing and editing of the commentary.

## CONFLICT OF INTEREST STATEMENT

The authors declare no conflicts of interest.
